# Immune Diseases: Challenges, Hopes and Recent Achievements

**DOI:** 10.3390/ph17010097

**Published:** 2024-01-11

**Authors:** François Dufrasne

**Affiliations:** Microbiology, Bioorganic and Macromolecular Chemistry Unit, Faculty of Pharmacy, Université Libre de Bruxelles (ULB), Boulevard du Triomphe, 1050 Brussels, Belgium; francois.dufrasne@ulb.be

Although they have been greatly described for about 50 years, we have gained a much greater understanding of immune diseases since the beginning of this millennium. The main reasons are as follows:

(i) A better comprehension of the intricate mechanisms involved in immunology [1].
(ii) The decoding of the human genome together with the mechanisms behind the epigenetic regulation of gene expression that shed light on the relationships between genes and immune diseases, together with their heritability [2,3,4].
(iii) The improvement in the diagnostic tools available for physicians and researchers that allow us to better define immune diseases, their stages and hallmarks [5].

This family of diseases is continuously attracting much attention from the medical world. The reason is that their prevalence has clearly increased during recent decades, and furthermore, immunological mechanisms have been proposed to be at the origin of several diseases with which a relationship was not initially obvious (for example, some cardiovascular—hypertension included—and psychiatric diseases). Consequently, the role of immunity in human health is pivotal and will still need many investigations in the future.

As many professionals know, treating immune diseases is not an easy task. When immunity is deregulated, dampening or suppressing it is logical but also especially dangerous for the patient. The expected side effects of immunomodulators and, more obviously, of immunosuppressants are opportunistic infections and secondary cancers. However, due to the immune system’s connection to other physiological systems in the body, many other effects can appear such as metabolic, cardiovascular and neurological events [6]. Attempts to act selectively on one mechanism responsible for the disease rather than on the whole immune system is thus crucial, and many pharmaceutical companies are striving to meet this challenge. On the contrary, in some diseases, it is necessary to increase immune defences, as for cancers and viral infections. In those cases, the use of biological drugs such as antibodies and interferons are the best options, the former having generally less side effects than the latter. However, since complete specificity for targeted cells cannot be achieved, some adverse effects will occur inevitably.

Despite the numerous questions still without satisfactory answers, much progress in the development of new drugs has been made recently, including among them the following:

- The discovery of immune checkpoint inhibitors in cancer therapy [7].
- JAK inhibitors in arthritic and inflammatory bowel diseases [8].
- Sphingosine-1-phosphate receptor modulators in multiple sclerosis [9].
- The development of disease-modifying therapies (DMT) in arthritic immune diseases [10].
- The marketing of “imide” drugs as immunomodulators against multiple myeloma [11].

Describing the mechanisms behind immune diseases and their implications for finding new drugs is an enormous task, far beyond the scope of this Special Issue. However, this selection of papers, focusing on some aspects of the immune system and molecules acting on it, will shed light on some interesting and sometimes unexpected discoveries. We hope that researchers find herein useful information for their work and that medical practitioners can improve their knowledge about a field of growing importance.
